# Emmerich’s So-called “Naples Cholera Bacteria”*From an address delivered before the Medical Society of Munich, April 1, 1885. (Taken from the “Berliner Klinische Wochenschift,” 1885, p. 237.)

**Published:** 1885-10

**Authors:** 


### Art. XXX.—EMMERICH'S SO-CALLED "NAPLES CHOLERA BACTERIA." *

The cholera question is still the one that is at present absorbing most of the attention of the world's patho-mycologists. The discovery of Koch's comma has awakened more discussion than any other of the kind that has ever been made. While but few, if any, observers deny its presence in connection with Asiatic cholera, and in fact, many of the most competent of them fully agreeing with Koch—such as Van Ermingam of Brussels, Watson, Cheyne—others have not accepted his conclusions, even going so far as to deny its diagnostic value when cultivated in tubes of flesh-water gelatine, which is misleading and dangerous in the light of the almost universal testimony to the contrary. Of this latter class is Klein—an English observer, in whom very little reliance can be placed, if we are to judge from the trustworthiness of any work of this kind that has yet emanated from him.

The most interesting, and upon its face, most trustworthy observations in opposition to those of Koch and his supporters that have as yet been given to the public have proceeded from Dr. Emmerich, a doctor of Munich, attached to the Biological Laboratory of the University at that place. Emmerich was sent by the government to Naples during the eruption of cholera in that city in the fall of 1884, in order to get material for bacteria investigations. He went after Koch's comma in particular and to make all the study of disease possible *in loco*. It might be here mentioned that one of the great incentives to opposition to Koch's observations and conclusions was the sacredness with which he guarded his culture material, no one being able to get any from him, and the exclusiveness

* From an address delivered before the Medical Society of Munich, April 1, 1885. (Taken from the "Berliner Klinische Wochenschift," 1885, p. 237.)

which want of room and other unavoidable circumstances threw about the cholera courses at the laboratory of the Imperial Board of Health at Berlin, during the last winter, for which Koch was not to blame in any manner.

The observations which Emmerich made led him to such entirely different conclusions than those of Koch with reference to the specific bacteria of cholera, that they deserve the widest publicity, and most sceptical study, especially as we have that dreaded disease here this summer.

At the meeting above alluded to, the remarks of Dr. Emmerich were essentially as follows :

It is at present the prevailing opinion among pathologists that the vibrio of Koch is the cause of Asiatic cholera, supporting themselves by the assumption that "no other form of pathogentic fungi are to be found in the blood or organs of cholera patients." My investigations at Naples have led me to quite the opposite conclusion, for in the blood *and organs* of nine cholera cases I *found the same form of bacteria*, which further study and repeated experiments have led me to strengthen my opinion that they must be looked upon as the immediate cause of cholera.

Emmerich has previously reported these bacteria, a staff-like (rod) bacteria twice as long as thick, somewhat resembling those of typhoid and not vibrios or curved bacteria. The vibrios of Koch can be of but secondary importance, as they have only been found in the intestine (or discharges) and but exceedingly seldom, and in the walls of the intestines, and never in the organs of diseased persons. It may yet be possible to demonstrate that the Koch comma is a constant resident in the intestinal canal of man, or one which developes there only under the peculiar conditions present during cholera.

As I have already mentioned, Emmerich's first examinations and experiments have found endorsement by others, subsequently performed, in which V. Schlen and Buchner have taken part. The first question to decide is, "Do the fungi found in cholera cadavers occur as pure cultivations in the blood and organs? or have we to do with a mixture of various forms?" The first would speak for their pathogenetic importance, while the last would have the contrary value. Peate cultivations made in Munich with the material from the tube cultivations inoculated direct from the blood and organs of cholera patients in Naples have abundantly shown that the fungi were present in an unadulterated condition. For the present I will designate them as the "Naples cholera bacteria."

As further proof of a most convincing character of the purity of these Naples cholera bacteria in the organs of cholera cadavers has been given "by the studies of V. Schlen upon microscopic preparations from the portions of organs brought direct from Naples in absolute alcohol. In these organs, as well as in material brought from India, he was enabled to find these peculiar bacteria in such quantities and so widely dispersed as to sufficiently warrant their ætiological relation with the phenomena of cholera, as well as the pathological conditions observed in the organs of persons that have died of the disease." We have the right to assume that these bacteria are the ætiological movement in connection with cholera, that we have to think the typhus (typhoid) bacillus is the cause of that disease.

The constancy with which these bacilli were found in all tissues that were examined for them, naturally leads us to the question: "How is it that they have been overlooked by other investigators?"

This has not been the case, however. It is well known that Koch made no mention of the comma during his Egyptian studies, but did speak of finding short rod bacteria in the walls of the intestines, while the curved vibrio came first to his notice in India. The French Cholera Commission to Egypt also reported finding short rod bacteria in the walls of the intestines and in their contents.

The studies of Buchner with reference to the biological peculiarities of the Naples cholera bacilli have shown that they bear closer resemblance to those of typhus than any other known form. This conformity was not only in their microscopical appearance, but also in the group, or colony form, but especially in the chemical characteristics, while other bacteria of the human body, drinking water, etc., showed great variations from them in these essentials. These cholera bacteria have far more vital energy than those of typhus, which entirely corresponds with the clinical department of the respective diseases. The Naples bacilli are very resistant to external influences, possessing an exceeding degree of vitality; they readily withstand exposure to (minus)—24° C for a period of twelve days, as well as four weeks desiccation at room temperature. These peculiarities are directly opposed to those of Koch's bacteria, which will not withstand desiccating for over three or four hours, and especially to be noted is the fact that these Naples bacteria can live without oxygen, which is not the case with Koch's comma, and is not without value in considering them as an inficiens.

The most striking proof of the correctness of our conclusions has been given by our experiments upon animals, which resulted in producing disease phenomena in almost every essential corresponding to those of Asiatic cholera in man.

Thirty guinea pigs, four dogs, six cats and a monkey were used for this purpose. The material was introduced into most of them sub-cutaneously. Diarrhœa and vomiting did not occur in the guinea pigs, but did in a most severe form in the larger animals. One of the dogs had repeated attacks of emesis, and eighteen diarrhœal discharges, finally became rice-water-like, in twenty-four hours. Another dog vomited twelve times in the same period. The vomitus consisted at first of particles of food, and had a decided acid reaction. Later it became more watery and contained yellowish flakes of mucous which had an alkaline reaction, as is the case in man. The other clinical phenomena, such as cyanosis, rigor, stupor, the rapid emaciation, anurias, indican in urine, etc., were also almost constant in the larger animals.

The chief conditions of value revealed by the microscopical examination of these animals were excessive rigor mortis, emaciation (not loss of fat), dry musculature, pleuræ and peritonitis, covered with a viscid mucous mass; ecchymosis in both; red color of anterior intestines, alkaline reaction of the thin, watery, or gruel-like, contents of the intestines, mesentery glands swollen, spleen always normal in size, liver dull red, gall and bladder much distended, kidneys of a dark brown-red color, yellowish striæ upon a grayish-red surface, were observable in these organs when the disease had two or three days duration; bladder either empty or contained a few drops of clouded urine. Above all these conditions, especial emphasis must be placed upon the constantly appearing viscid mucous peritoneal exudate, in every form of inoculation, which, as is known, is so peculiarly characteristic of human cholera.

The Naples bacteria were present in this exudate in immense quantity.

Are they also present in such numbers in this same material in human cholera?

The organs of all these animals were also proven in the same manner as those from human beings in Naples:

1st. By cultivations.

2d. By microscopic examinations, the results entirely confirming those previously alluded to.

The Naples bacteria were also found in the contents of the intestines. This is of importance, as it shows for the first time that the sub-cutaneously introduced bacilli not only find their way into the blood and organs, but also into the intestinal cavity. This fact does more to shake the drinking water theory than anything else. Feeding animals with great quantities of the bacilli gave negative results in each case. The same animals at once reacted in the above described manner when subjected to sub-cutaneous inoculation.

The autopsy of the monkey was made with great care by Prof. Bollinger. One of the most essential pathological phenomena was that held by Piragoff, Virchow, and lately, Eichhorst, as characteristic of cholera, and that is the typhus-like changes in the ileum with beet-like—"beetætige"—swelling of Peyer's plaques with a dark red area around them. The omentum, mucous of stomach and pericardium were the seat of numerous ecchymosis; spleen normal, beginning fat degeneration in the kidneys. With reference to the results in the intestines it can only be said that if we will not accept them as due to cholera, we must fall back on the assumption that we had to do with an exceedingly violent and acute case of typhus abdominalis (typhoid). All the accompanying phenomena contradict this last assumption as well as the circumstance that no typhus, but quite another bacillus was injected, which we have also demonstrated, in the organs and blood. There remains to us, then, but one conclusion, viz.: the acute infectious disease of which the monkey in question died must be looked upon as genuine Asiatic cholera.

These Naples cholera bacteria have another essentially varying quality from other fission fungi, *i.e.*, those of septicæmia, in that it requires a proportionally great number of them to cause infection in an animal. Small quantities are absolutely inactive ; their virulence is not increased by continued direct inoculation through a series of animals. The want of any disposing conditions in animals to cholera is probably the reason that such large quantities are required to produce infection.

Emmerich sums up the results of his various investigations and experiments as follows :

I consider the Naples cholera bacteria as the active agent which we must make answerable for the pathological changes in cholera. Koch's theory fails of proof and is supported chiefly by hypothesis. Koch says that "he assumes that the comma bacilli are to be demonstrated early in the disease. The want of resistability of Koch's vibrio, the fact that they lose their vitality so quickly on desiccation is directly opposed to the epidemiology of cholera, which in India occurs during the time of the greatest drought. Finally, the vibrio of Koch are not always present in predominating numbers in the alvine discharges of cholera patients. I have often made ten or more plate cultures from one discharge during my stay in Naples and found none. On the contrary, the Naples bacteria have always been present, frequently in nearly pure cultivations, and always predominating over the vibrio of Koch in number."

I do not, by any means, mean to assert that these investigations into the etiology of cholera have entirely closed our work, but do think that they have given us a firm foundation from which to proceed in the work.

So much for Emmerich. V. Schlen, Pettenhoffer, Frolenius, Buchner and others all took an active part in the discussion, to the original report of which we refer those interested, as we have here only essayed to give the direct evidence brought forward by Emmerich, and the conclusions to which they have led. The gentlemen named above all bear witness to the correctness of his assertions.

